# Supplementary material for: Lightning Pose 3D: an uncertainty-aware framework for data-efficient multi-view animal pose estimation
Source: bioRxiv. 2026 Apr 23:2026.04.20.719731. Preprint. [Version 1] doi: 10.64898/2026.04.20.719731 (PMC13131684; doi:10.64898/2026.04.20.719731)
Supplement: Supplement 1 [file NIHPP2026.04.20.719731v1-supplement-1.pdf]

bioRxiv preprint doi: https://doi.org/10.64898/2026.04.20.719731; this version posted April 23, 2026. The copyright holder for this preprint (which was not certified by peer review) is the author/funder, who has granted bioRxiv a license to display the preprint in perpetuity. It is made available under aCC-BY 4.0 International license.

8 Supplementary Video Captions

Supplementary Video 1. Predictions from Ensemble Median, linear mvEKS, and linear mvEKS with variance inflation for the paw1LH and paw2LF keypoints, shown with error bars indicating uncertainty in the x and y directions, for the Treadmill Mouse dataset. Orange error bars indicate ensemble variance; green error bars indicate posterior variance from mvEKS. This session corresponds to the example shown in Extended Data Fig. 4a-b for paw1LH.

Supplementary Video 2. Baseline of ResNet-50 versus LP3D + mvEKS with variance inflation (200 labeled frames) for both paws, IBL dataset, similar to the example session in Fig. 4e. Red error bars indicate ensemble variance; green error bars indicate posterior variance from mvEKS. At frames 1070–1077 and frame 1084, LP3D + mvEKS outperforms ResNet-50, maintaining consistent tracking across views during occlusion events where ResNet-50 produces erroneous predictions. At frames 10188–10195, ResNet-50 outperforms LP3D + mvEKS, though both models produce reasonable predictions in this case.

Supplementary Video 3. LP3D (200 labeled frames) + mvEKS with variance inflation, Fly dataset. Error bars indicate posterior variance from mvEKS.

Supplementary Video 4. LP3D (200 labeled frames) + mvEKS with variance inflation, Chickadee dataset. Error bars as in Supp Video 3.

Supplementary Video 5. LP3D (200 labeled frames) + mvEKS with variance inflation, cropped on the black mouse from the Resident-Intruder dataset. Error bars as in Supp Video 3.

Supplementary Video 6. LP3D (200 labeled frames) + mvEKS with variance inflation, cropped on the white mouse from the Resident-Intruder dataset. Error bars as in Supp Video 3.

Supplementary Video 7. Comparison of DANNCE and LP3D model predictions on Fly dataset alongside ground truth labels (150 test frames) for 12 keypoints (2 medial joints per leg across all 6 legs), corresponding to the results shown in Extended Data Fig. 1c.

Supplementary Video 8. Comparison of DANNCE and LP3D model predictions on the Chickadee dataset alongside ground truth labels (143 test frames) for the left foot, right foot, top of head, and tail base keypoints, corresponding to the results shown in Extended Data Fig. 1c.

bioRxiv preprint doi: https://doi.org/10.64898/2026.04.20.719731; this version posted April 23, 2026. The copyright holder for this preprint (which was not certified by peer review) is the author/funder, who has granted bioRxiv a license to display the preprint in perpetuity. It is made available under aCC-BY 4.0 International license.

9 Supplementary material

	Dataset				
Hyperparameter	Treadmill Mouse	Fly	Chickadee	IBL	Resident-Intruder
config.data					
image_resize_dims	256×256				
num_keypoints	7	30	18	3	22
config.model					
backbone	vits_dino				
model_type	heatmap_multiview_transformer				
head	heatmap_cnn				
heatmap_loss_type	mse				
config.training					
optimizer	Adam				
optimizer_params.learning_rate	5e-5				
lr_scheduler_params.multisteplr.gamma	0.5				
lr_scheduler_params.multisteplr.milestones	[2000, 3000, 4000]				
train_batch_size	8				
train_prob / val_prob	0.95 / 0.05				
imgaug	dlc				
imgaug_3d	false	true	true	true	true
patch_mask.init_step	700				
patch_mask.final_step	5000				
patch_mask.init_ratio	0.1				
patch_mask.final_ratio	0.5				
unfreezing_step	400				
max_steps	5000				
config.losses					
supervised_reprojection_heatmap_mse.log_weight	—	3	3	3	3
config.callbacks					
anneal_weight.freeze_until_epoch	—	60	60	60	60

Table 1: **Training hyperparameters by dataset.** All datasets share the same architecture (multi-view transformer with CNN head), optimizer (Adam), and most training settings. Dataset-specific differences are highlighted where applicable. Entries correspond to parameter fields in the standard Lighting Pose model configuration file (see example at `https://github.com/paninski-lab/lightning-pose/blob/v2.0.8/scripts/configs/config_default_multiview.yaml`).

bioRxiv preprint doi: https://doi.org/10.64898/2026.04.20.719731; this version posted April 23, 2026. The copyright holder for this preprint (which was not certified by peer review) is the author/funder, who has granted bioRxiv a license to display the preprint in perpetuity. It is made available under aCC-BY 4.0 International license.

		Decoding R^2	Decoding error (mm/s)
IBL 3D paw speed (left)	ResNet-50	0.185±0.013	0.53±0.06
	LP3D	0.208±0.012	0.51±0.06
	LP3D + mvEKS	0.260±0.015	0.32±0.04
IBL 3D paw speed (right)	ResNet-50	0.209±0.012	0.48±0.05
	LP3D	0.216±0.011	0.41±0.04
	LP3D + mvEKS	0.287±0.013	0.30±0.03

Table 2: **Neural decoding performance in IBL data across pose estimation models.** Values are mean ± SEM across $n = 39$ sessions. Decoding R^2 is the coefficient of determination; decoding error is the mean absolute error (MAE) in mm/s.

bioRxiv preprint doi: https://doi.org/10.64898/2026.04.20.719731; this version posted April 23, 2026. The copyright holder for this preprint (which was not certified by peer review) is the author/funder, who has granted bioRxiv a license to display the preprint in perpetuity. It is made available under aCC-BY 4.0 International license.

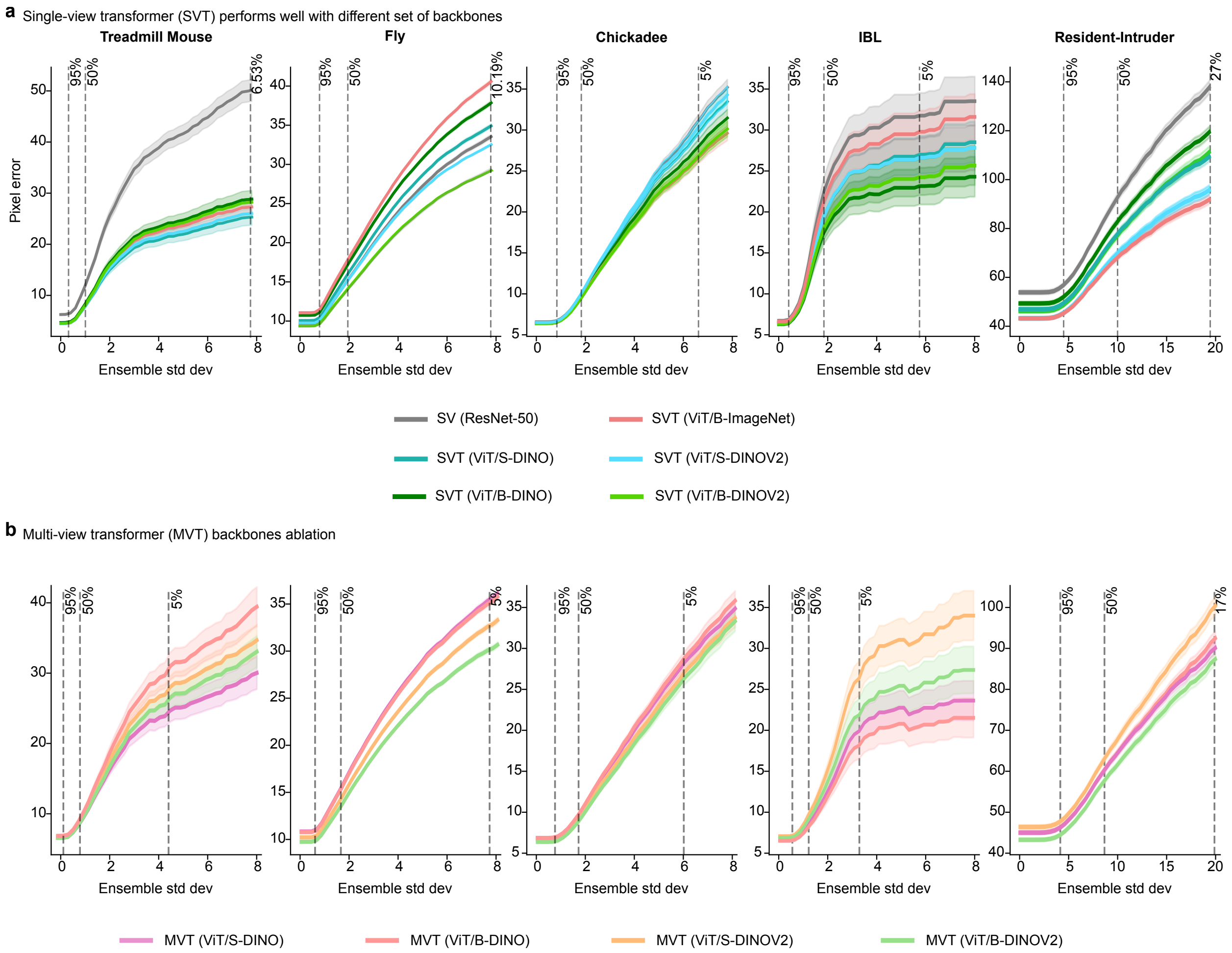

Supplementary Figure 1: **Comparison of pretrained transformer backbones and ResNet-50 for SVT and MVT. a,** Comparing single-view transformer (SVT) performance with different transformer pretrained backbones by showing pixel error as a function of keypoint difficulty (lower is better). Dashed vertical lines indicate the percentage of data used for the pixel error computation. VIT/B is a "base" model (∼80M parameters), VIT/S is a "small" model (∼20M parameters); ResNet-50 has ∼20M parameters. ViT-B/ImageNet refers to ViT B-16 pretrained on ImageNet using masked autoencoders (MAE; He et al., 2022). DINO is a self-supervised pretraining method based on knowledge self-distillation with Vision Transformers (Caron et al., 2021); DINOv2 is an improved version trained on a larger curated dataset (Oquab et al., 2023). ViT-B/DINO and ViT-S/DINO denote base and small ViT models pretrained with DINO on ImageNet, respectively; ViT-B/DINOv2 and ViT-S/DINOv2 denote base and small ViT models pretrained with DINOv2. **b,** Comparing multi-view transformer (MVT) performance with different transformer pretrained backbones by showing pixel error as a function of keypoint difficulty.

bioRxiv preprint doi: https://doi.org/10.64898/2026.04.20.719731; this version posted April 23, 2026. The copyright holder for this preprint (which was not certified by peer review) is the author/funder, who has granted bioRxiv a license to display the preprint in perpetuity. It is made available under aCC-BY 4.0 International license.

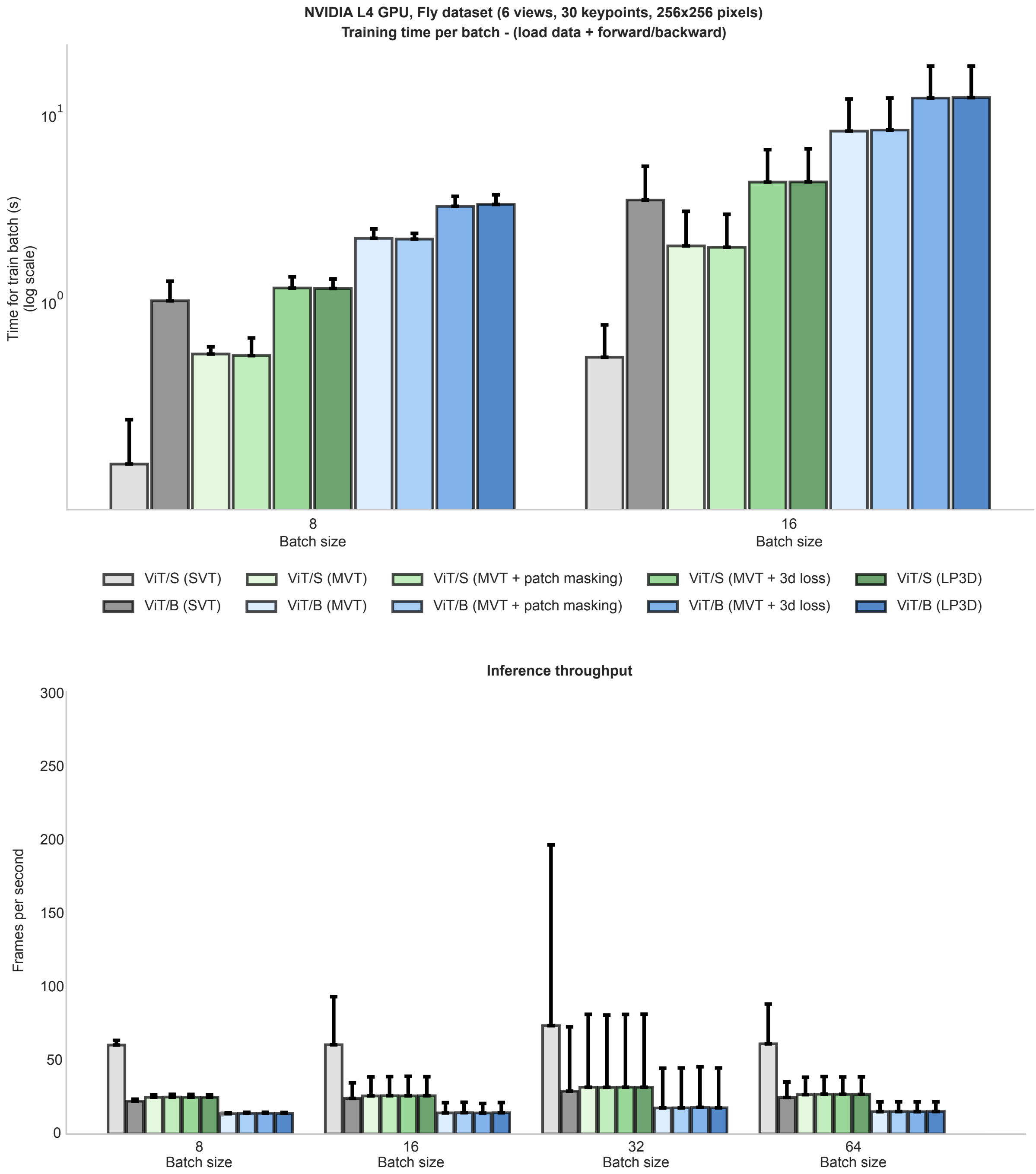

Supplementary Figure 2: **Training time per batch and inference throughput.** Timing benchmarks are performed with the Fly dataset on an entry-level NVIDIA L4 GPU. Top panel: each bar depicts the mean batch processing time (in seconds) and 95% CI over $n = 100$ batches; y-axis is log-scaled. Bars are grouped by batch size (8/16) along the x-axis. Bottom panel: each bar depicts the mean frames per second with 95% CI over $n = 100$ batches. Bars are grouped by batch size (8/16/32/64). For both panels, 50 warm-up batches were processed before recording measurements.

bioRxiv preprint doi: https://doi.org/10.64898/2026.04.20.719731; this version posted April 23, 2026. The copyright holder for this preprint (which was not certified by peer review) is the author/funder, who has granted bioRxiv a license to display the preprint in perpetuity. It is made available under aCC-BY 4.0 International license.

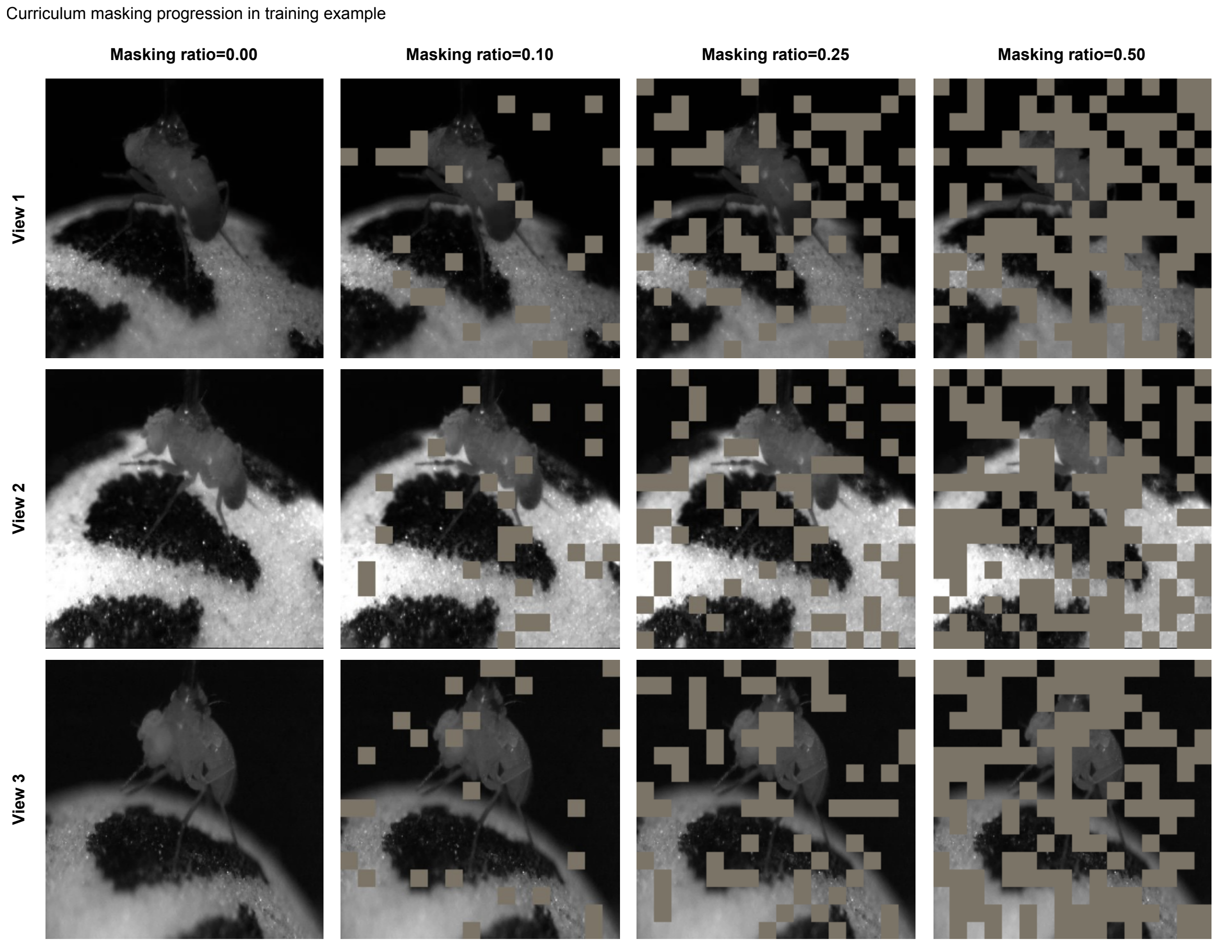

Supplementary Figure 3: **Simulating occlusions in multi-view transformer training via curriculum patch masking.** Visualization of the patch masking scheme applied to three example camera views from the Fly dataset. Each 256×256 input image is divided into a regular grid of 16×16 pixel patches, and a fraction of patches are randomly zeroed out (brown regions). Columns show increasing masking ratios (0.00, 0.10, 0.25, 0.50) corresponding to the curriculum schedule used during training: the model first trains without masking, then the masking ratio is linearly increased from an initial to a final value over a defined range of training steps. By synthetically occluding portions of individual views, this scheme simulates the partial occlusions that frequently arise during natural animal behavior, forcing the model to leverage cross-view information through the self-attention mechanism of the multi-view transformer. The model thus learns to compensate for missing information in one view by drawing on unoccluded views, improving robustness to occlusions at inference time.

bioRxiv preprint doi: https://doi.org/10.64898/2026.04.20.719731; this version posted April 23, 2026. The copyright holder for this preprint (which was not certified by peer review) is the author/funder, who has granted bioRxiv a license to display the preprint in perpetuity. It is made available under aCC-BY 4.0 International license.

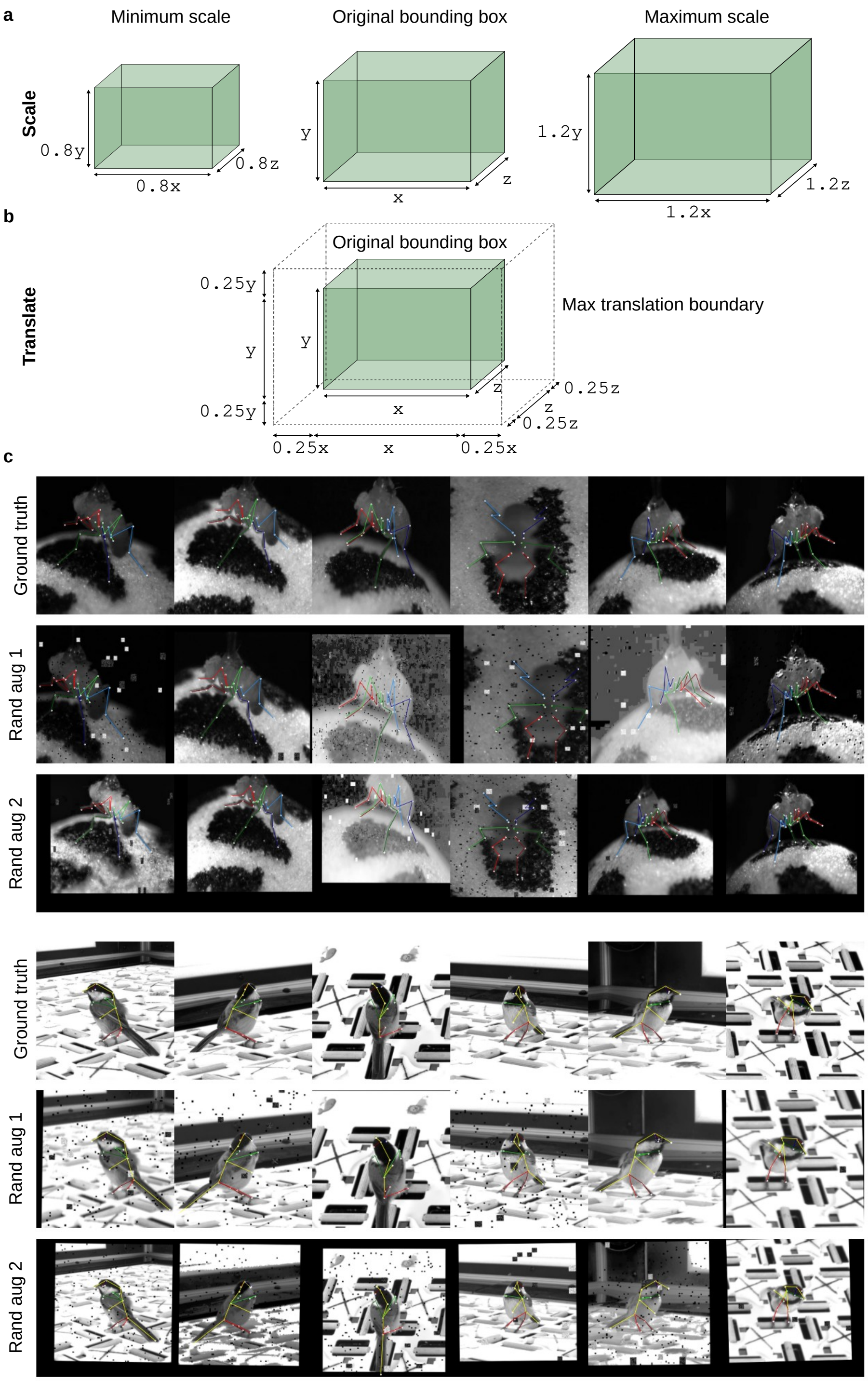

Supplementary Figure 4: **Illustration and examples of 3D augmentations. a,** The *scale* augmentation samples a random scalar from $[0.8, 1.2]$ and multiplies each centered keypoint by this value. This resizes the 3D bounding box while preserving the subject's aspect ratio and maintaining the centered position. **b,** The *translate* augmentation samples random scalars from $[-0.25, 0.25]$ for each dimension, then multiplies these by the corresponding bounding box dimension length. This produces translation amounts that scale appropriately with subject size. **c,** Augmentations for datsets with camera calibration parameters combine scale and translation in the 3D space with view-independent appearance augmentations (e.g., pixel noise and brightness).

bioRxiv preprint doi: https://doi.org/10.64898/2026.04.20.719731; this version posted April 23, 2026. The copyright holder for this preprint (which was not certified by peer review) is the author/funder, who has granted bioRxiv a license to display the preprint in perpetuity. It is made available under aCC-BY 4.0 International license.

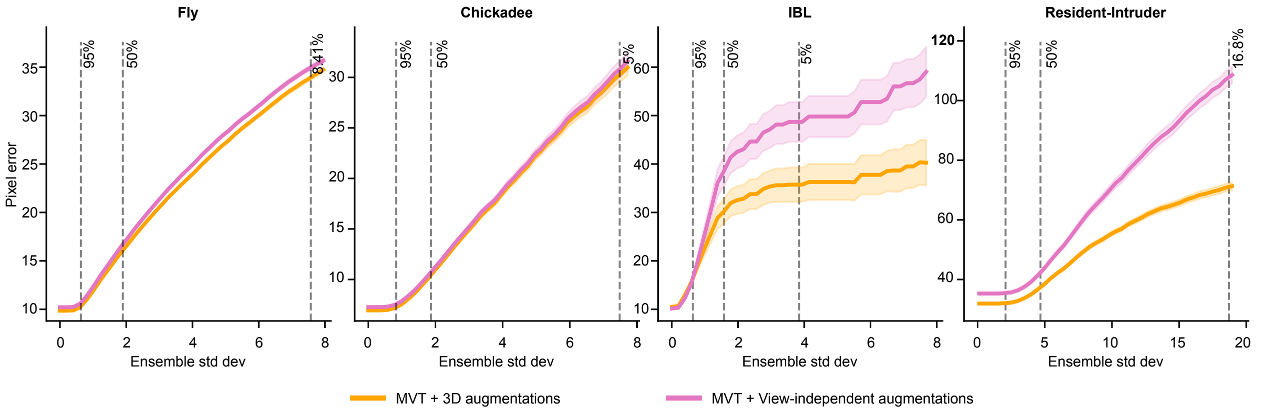

Supplementary Figure 5: **3D augmentations compare similarly or outperform view-independent augmentations.** 3D augmentations, described in (Supp Fig. 4), can be applied to any dataset with camera calibration parameters. These geometrically consistent augmentations perform similar to or better than view-independent geometric augmentations, such as rotations and scales. Both forms of augmentation use the same appearance-based augmentation pipeline (pixel noise, brightness/contrast manipulations, etc.).

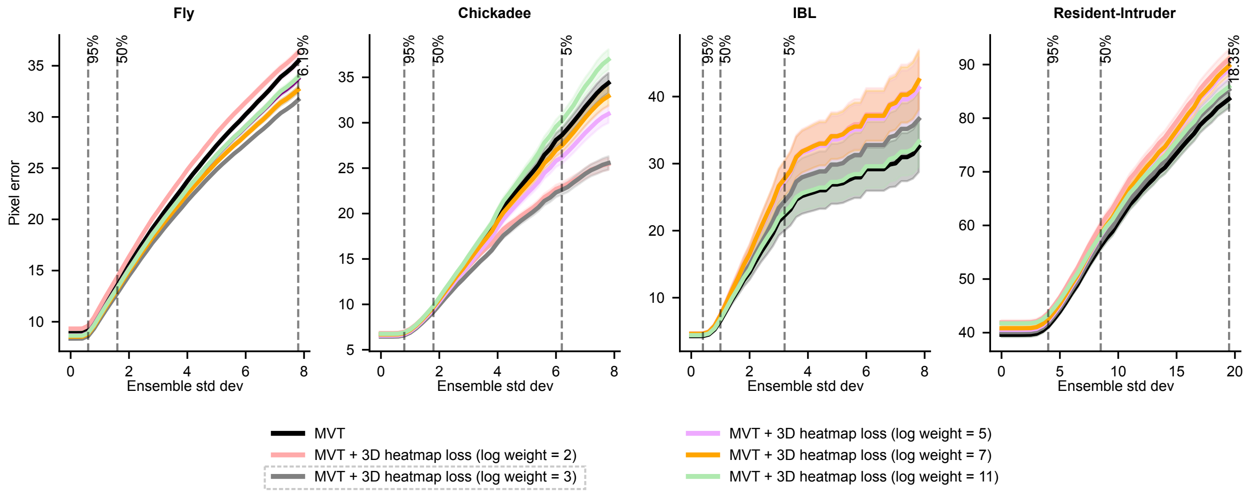

Supplementary Figure 6: **Hyperparameter selection for the 3D reprojection loss weight.** Performance across datasets for different log weight values balancing the 3D reprojection loss against the 2D heatmap loss. Higher log weight values correspond to lower loss weights. The gray dashed line marks the selected value (`log weight=3`), which performs consistently well across all datasets and is used for all experiments.

bioRxiv preprint doi: https://doi.org/10.64898/2026.04.20.719731; this version posted April 23, 2026. The copyright holder for this preprint (which was not certified by peer review) is the author/funder, who has granted bioRxiv a license to display the preprint in perpetuity. It is made available under aCC-BY 4.0 International license.

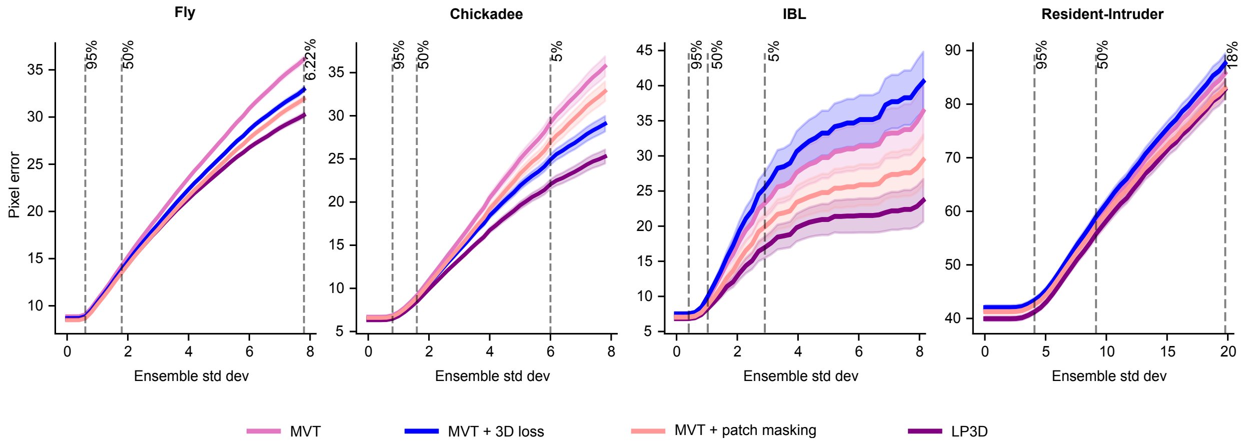

Supplementary Figure 7: **Patch masking and 3D reprojection loss provide complementary performance benefits.** Performance across all datasets, comparing MVT alone; MVT with patch masking; MVT with the 3D reprojection loss; and the full model with both patch masking and 3D reprojection loss (LP3D). For Fly and Chickadee, each component independently improves over MVT alone. For IBL and Resident-Intruder, the 3D loss alone hurts performance relative to MVT, yet combining it with patch masking surpasses patch masking alone, demonstrating that the two components are complementary.

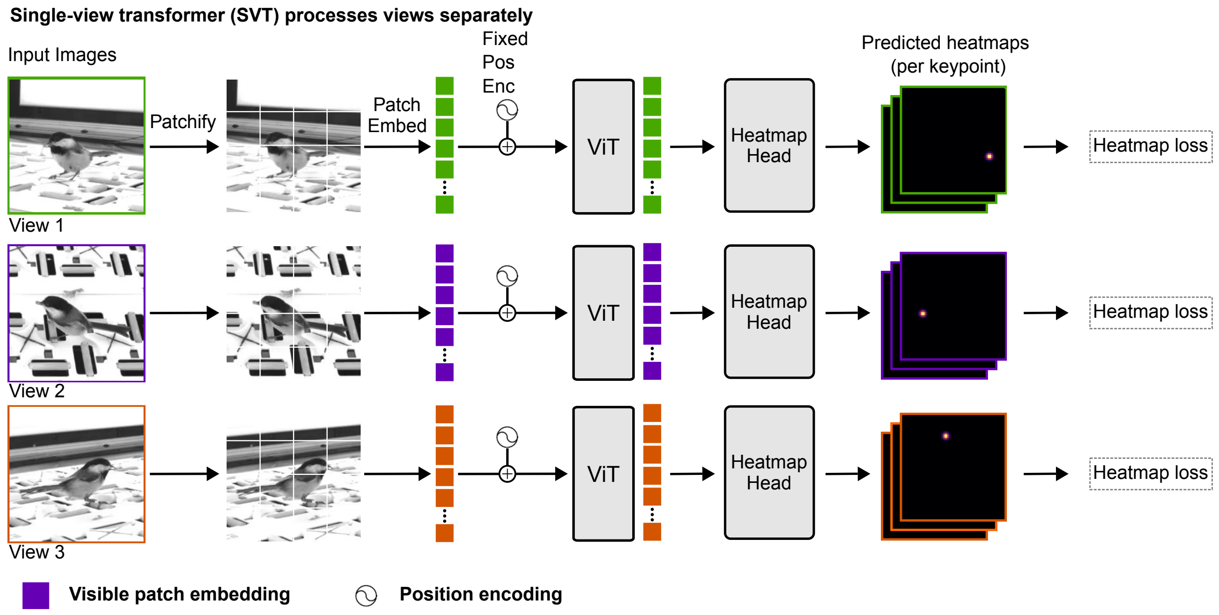

Supplementary Figure 8: **Single-view transformer (SVT) architecture.** Input frames are split into patches, embedded into a latent space, combined with a fixed position encoding, and processed through a vision transformer (ViT). Outputs are reshaped and passed to a heatmap head. The model is trained with a mean square error (MSE) loss between predicted and ground truth heatmaps. Multiple views are processed independently.

bioRxiv preprint doi: https://doi.org/10.64898/2026.04.20.719731; this version posted April 23, 2026. The copyright holder for this preprint (which was not certified by peer review) is the author/funder, who has granted bioRxiv a license to display the preprint in perpetuity. It is made available under aCC-BY 4.0 International license.

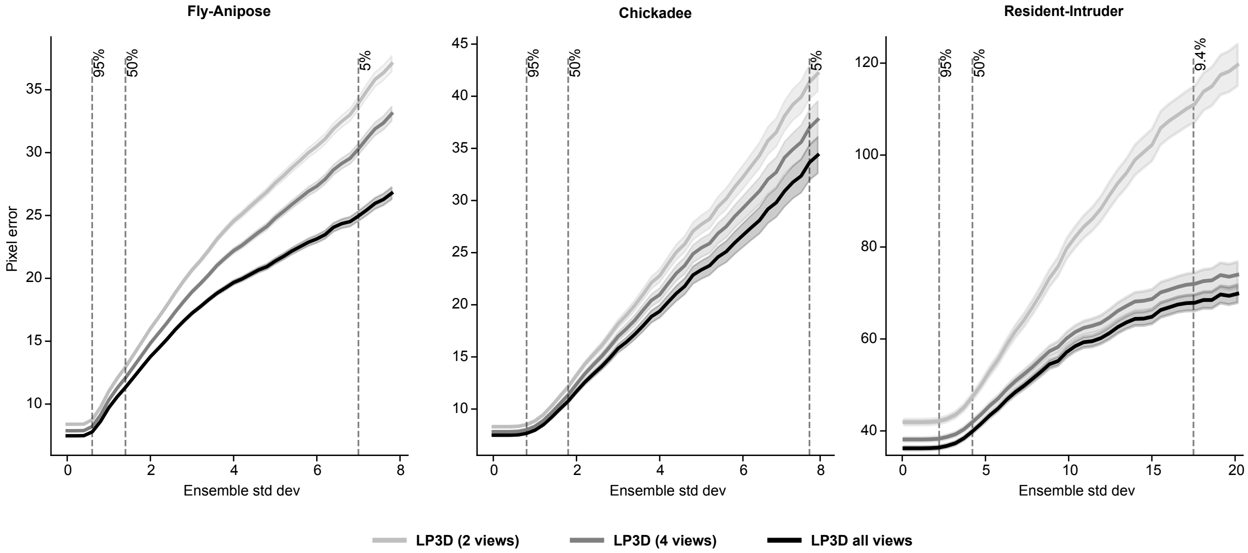

Supplementary Figure 9: **The accuracy of LP3D increases with the number of camera views.** The LP3D model was trained on the Fly and Chickadee datasets (both six-view) and on the Resident-Intruder (five-view). We varied the number of views used during training to either two, four, or five/six views (three random seeds per condition). The two views used for the two-view condition were a random subset of the four views, which in turn were a random subset of all available views. All models were evaluated on the same shared two-view subset for 2D keypoint prediction (these two views are present in each view subset). This design demonstrates that increasing the number of views used during training significantly improves the LP3D model's performance, as evaluation is held constant across all conditions.

bioRxiv preprint doi: https://doi.org/10.64898/2026.04.20.719731; this version posted April 23, 2026. The copyright holder for this preprint (which was not certified by peer review) is the author/funder, who has granted bioRxiv a license to display the preprint in perpetuity. It is made available under aCC-BY 4.0 International license.

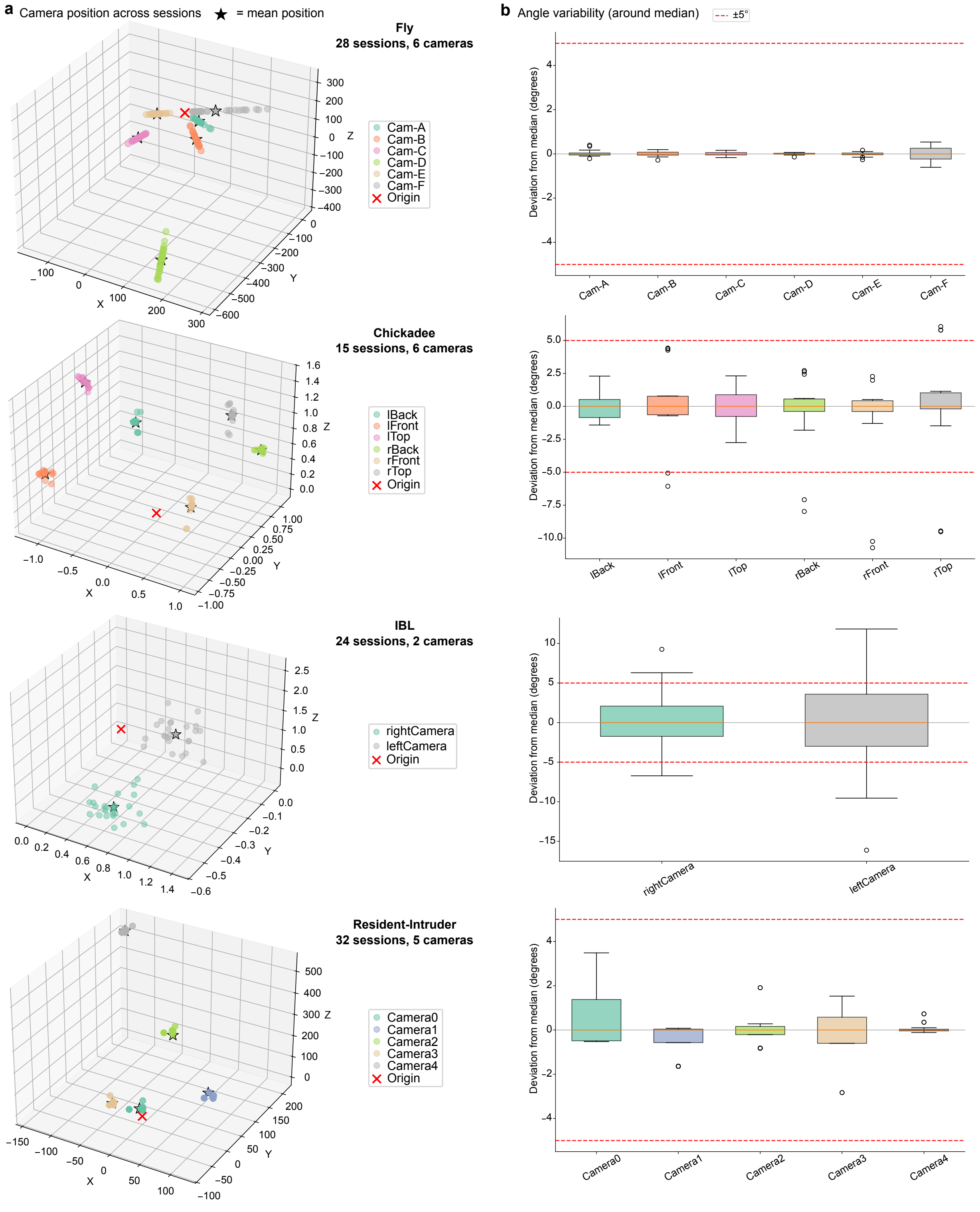

Supplementary Figure 10: **Camera stability across recording sessions. a,** Three-dimensional positions of each camera's optical center, estimated from per-session calibration files, for four benchmark datasets (Fly, Chickadee, IBL, Resident-Intruder). Each point represents one session; stars indicate the per-camera mean position. **b,** Deviation of each camera's rotation angle (Rodrigues vector norm) from its per-camera median across sessions. Dashed red lines mark a $\pm 5^\circ$ stability threshold. Across all datasets, cameras exhibited minor session-to-session displacement and orientation drift, yet pose estimation accuracy remained consistent, demonstrating that our pipeline is robust to the rig perturbations typical of real experimental settings.
